# An Evaluation of Parent and Child Perceptions About Pediatric Pedestrian Safety Skills

**DOI:** 10.1007/s10566-026-09971-y

**Published:** 2026-08-12

**Authors:** Casie H. Morgan, Lindsay M. Stager, Cierra Hopkins Smith, Leslie A. McClure, Angelina R. Davis, Anna Johnston, Jiabin Shen, Yixin Wang, David C. Schwebel

**Affiliations:** 1 Medical University of South Carolina, Charleston, United States; 2 Brown University, Providence, United States; 3 Miriam Hospital, Providence, United States; 4 Kennedy Krieger Institute, Baltimore, United States; 5 Saint Louis University, St Louis, United States; 6 University of Massachusetts Lowell, Lowell, United States; 7 University of Alabama at Birmingham, Birmingham, United States; 8 University of Iowa, Iowa City, United States

**Keywords:** Unintentional injury, Safety, Children, Perception, Pedestrian injury risk

## Abstract

**Background:**

Pediatric pedestrian injury is a major public health concern. Children’s perceived abilities correlate with behavior across many pediatric behavioral health contexts, but this association is untested regarding pedestrian safety.

**Objective:**

If children’s perceptions relate to behavior in pedestrian settings, there are implications for targeted intervention. We evaluated associations between parent and child perceptions of children’s street-crossing ability with actual abilities within a virtual reality (VR) pedestrian platform.

**Methods:**

As part of a larger clinical trial, 497 children aged 7–8 years participated in baseline pedestrian skill assessments and subsequent virtual reality training sessions. Training terminated when children achieved adult competency. Outcomes included baseline unsafe crossings and number of visits to reach adult competency. Baseline perceptions of children’s ability were assessed via parent and child self-report. Linear regression models were completed for each outcome, with parent perception, child perception, child age, and child sex as predictors.

**Results:**

The first linear regression demonstrated significant associations between baseline unsafe crossings and both parent perception of real-world crossings (β=−0.10771, *p*<.05) and child age (β=−0.18288, *p*<.01). A second linear regression examining the number of training visits required to achieve adult competency also identified significant associations between parent perception of real-world crossings (β=−0.13499, *p*<.01) and child age (β=−0.13927, *p*<.01).

**Conclusions:**

Parents’ perceptions of children’s abilities are significantly associated with children’s pedestrian skills, whereas children’s own perceptions show less consistent and non-significant associations. Prioritizing youth voices alongside educating parents about pedestrian safety risks may offer complementary and effective strategies to support children to become safe, active pedestrians.

**Clinical Trial Registration:**

The trial was registered at ClinicalTrials.gov, Identifier NCT02948400.

## Introduction

Pediatric pedestrian injury is a major global public health concern (Kendi et al., 2023). In the United States, an estimated 509 children under the age of 19 were fatally injured in pedestrian crashes in 2023, and an additional 29,211 children visited the emergency department due to an unintentional nonfatal pedestrian injury (CDC, 2025).

The United Nations Convention of the Rights of the Child (UNCRC) acknowledges children’s right to participate in decisions and actions that impact their lives, including their safety (United Nations, 1989). Article 12 of the UNCRC emphasizes that children have the right to be listened to and taken seriously, with their evolving capacities considered (United Nations, 1989; Children and Young People’s Commissioner Scotland, n.d.). Efforts to prevent pediatric unintentional injuries often tilt toward reliance on adults to protect children and mitigate harm, recognizing adults have more advanced cognitive skills and therefore may be viewed as protectors (Huynh et al., 2017). However, a focus on the child’s ability to maintain their own safety, especially as they grow older, and an integration of the adult-child dyad unit to reduce injury risk, may be more productive to understand and create the safest environments for children. In fact, extensive research demonstrates that effective child pedestrian injury prevention strategies are multifaceted, and efforts to target both adult supervisors and children themselves, as well as targeting other relevant factors such as environmental risk and driver behavior, are likely to yield the best protection for children on global roadways (Kendi et al., 2023; Schwebel et al., 2014).

A key aspect of promoting child pedestrian safety that acknowledges both adult and child agency is recognition of the child’s actual skills and ability (Barton & Schwebel, 2007). If acting without adult support, children must use their own perceived ability to decide which streets to cross and when to cross (Meir et al., 2013; O’Neal et al., 2018). Concomitantly, supervising adults, such as parents, must judge their children’s skill level to determine both the extent of independence permitted and the supervision required to keep their children safe (Morrongiello & Barton, 2009; Riaz et al., 2022). These decisions are most meaningful if perceptions about the child’s street-crossing ability, by adults and children respectively, are aligned both with each other and with the child’s actual skill level (Morrongiello & Corbett, 2015).

Engaging in roadways as a skilled pedestrian involves both developing the motor skills needed to cross the street independently and mastering advanced cognitive and perceptual competencies. Before and during the act of crossing the street, pedestrians engage in sophisticated cognitive processes, including perceiving environmental cues, evaluating the dynamic risks posed by oncoming traffic as well as the static features of the roadway, making rapid decisions, and initiating the motor actions required to cross (Morgan et al., 2023). Therefore, the act of judging a child’s pedestrian safety abilities must consider a child’s motor and cognitive-perceptual skillsets.

Children’s perceived abilities are associated with actual behaviors across many other pediatric behavioral health contexts, though the accuracy and precision of those perceptions vary (Niemistö et al., 2022; Putnick et al., 2020). Research on physical activity, for example, demonstrates that younger children tend to overestimate their own motor competence and choose to engage in physical activity (e.g., sports) beyond the limits of their actual ability (den Uil et al., 2023; Field et al., 2024; Morano et al., 2020). Conversely, children who underestimate their competency are more likely to disengage from activities despite having actual skills that would enable them; such disengagement may hinder further development and learning (Field et al., 2024; Morano et al., 2020; Niemistö et al., 2022). Research confirms that children’s perception of their own ability is dictated to some degree by age and development, with older and more developed children showing better ability to judge their own skills (Babic et al., 2014; den Uil et al., 2023; Plumert, 1995). This is promising from a safety education perspective, as children’s developing awareness can be engaged as a resource in safety education.

Related fields also suggest that the accuracy of parents’ perceptions of their children’s abilities varies across health domains (e.g., Lineweaver et al., 2022; Putnick et al., 2020). Parent reports of their school-aged children’s fundamental object control and locomotor movement skills, for example, are associated with their children’s actual skills (Maher et al., 2018). In other domains, parents have poor accuracy in assessing their child’s competencies. This is especially common for complex motor activities. For example, parents are consistently inaccurate in estimating their children’s swimming ability (De Pasquale et al., 2021; Morrongiello et al., 2014).

### Current Study

Guided by theory in child development and health behavior, this study examined associations between parent perception of children’s pedestrian ability, child perception of their own pedestrian ability, and children’s actual pedestrian abilities, as assessed in a virtual reality (VR) environment. The research was conducted among a sample of seven- and eight-year-old children engaged in a larger randomized clinical trial evaluating child pedestrian safety training. We posed three hypotheses: (1) children’s perceptions about child pedestrian ability would be associated with their actual skill; (2) parents’ perceptions about child pedestrian ability would be associated with their child’s actual skill; and (3) more advanced child age would be associated with greater pedestrian skills. Child sex was included as a covariate in our analyses due to previous findings suggesting sex differences in children’s cognitive (Pesce et al., 2018) and motor (Liong & Ridgers, 2015; Sando et al., 2024) skills.

## Method

### Participants

As part of a larger study (Schwebel et al. 2024a, b), 497 seven- and eight-year-old children were recruited from community sources. Exclusion criteria were minimal and included inability of the child and parent to communicate in English, prior study participation of a sibling, and physical or mental impairment prohibiting valid study participation. Parents of all participants provided informed consent to participate, and children provided informed assent. The research protocol was reviewed and approved by the Institutional Research Board at University of Alabama at Birmingham. The trial was registered at ClinicalTrials.gov, Identifier NCT02948400. There are no conflicts of interest to report. The primary author (CHM) and supervising author (DCS) take responsibility for the integrity of the data and accuracy of the data analyses.

### Procedure

#### Study Design

The larger study, a non-inferiority randomized clinical trial, evaluated whether children trained in a smartphone-based VR environment would develop pedestrian skills not inferior to children trained in a large, semi-immersive VR environment. Primary results (Schwebel et al. 2024a, b) are reported elsewhere; this report focuses on child and parent perceptions of child pedestrian ability at baseline assessment.

In the larger study, children participated in a baseline assessment and then were randomly assigned to one of two training groups. Training in both groups involved 30-minute training visits twice weekly until children reached adult levels of pedestrian safety or completed 25 training visits. A minimum of 6 training visits was required for all children.

Pedestrian behavior was assessed at baseline using a semi-immersive kiosk environment, which presents a three-sided view of a two-lane bi-directional street from a semi-immersive, first-person perspective on three monitors (details in Schwebel et al. 2024a, b). When the child feels it is safe to cross, they step down off a wooden curb to initiate crossing. At that point, the crossing becomes third-person and the child watches the safety of their crossing. At baseline assessment, participants crossed the virtual road 21 times. Seven crossings were in light traffic, defined as four vehicles per lane traveling at 25 miles per hour (mph), seven in moderate traffic (six vehicles per lane traveling at 30 mph) and seven in heavy traffic (six cars per lane traveling at 35 mph).

### Measures

#### Demographics

Child and family demographics were assessed by parent report.

#### Pedestrian Behavior: Unsafe Crossings

*Unsafe crossings* were defined as any collisions between the child and simulated vehicles, plus any “close call” instances when the child was within one second of being struck by an oncoming vehicle. The percentage of unsafe crossings across the 21 trials was calculated as an outcome variable by summing the number of hits and close calls and dividing by the total number of crossings across the three sets. For example, a child with three hits and four close calls had seven unsafe crossings across the 21 crossings, yielding a percentage of 33% of unsafe crossings. This construct represents the child’s performance in a pedestrian safety task.

#### Pedestrian Training: Learning Rate

All children were required to have at least 6 training visits, and training continued for up to 25 visits until adult competency was achieved. Adult competency was defined as crossing the virtual street safely over 95% of the time across two training visits with traffic speed and density matching those observed at the actual simulated site, a criterion based on previous research with > 300 adults. Just one child (0.2%) did not achieve adult competency by their 25th training visit, and data for that child were dropped from analyses involving this variable. The *number of training visits* required for children to achieve adult pedestrian competency was considered as an outcome measure in this study. This construct represents the child’s learning rate within the context of pedestrian safety.

#### Perception of Child Pedestrian Ability

Parents reported on their perception of their child’s real-world crossing ability via a self-report item: *Imagine your child is crossing a real street in a suburban area. There is a crosswalk but no traffic light. What percentage (0–100) of the time do you think your child would cross the street safely?*

Children reported on their perception of their own real-world crossing ability via a self-report item: *Imagine you are at a busy street. There is a crosswalk but no traffic light. Do you think you could get across the street safely?* Response options for the child question were on a five-point scale ranging from *1 – No*, *definitely not* to *5 – Yes*, *for sure*.

### Data Analysis

SPSS version 28 was used for all data analyses. Statistical assumptions were evaluated and satisfied for all variables. Missing data were evaluated using Little’s Missing Completely at Random (MCAR) test, which was non-significant, χ^2^(24) = 26.34, *p* =.336, suggesting the assumption of MCAR was met (Little, 1998). Thus, listwise deletion would not bias parameter estimates and was used to handle missingness. Missingness ranged from 0% (age, sex, unsafe crossings) to 8.4% (number of training visits). Preliminary analyses examined correlations between demographics, parent perception, child perception, and child pedestrian outcomes. Two multivariable linear regression models were fitted, one with unsafe crossings as the outcome variable and the other with the number of training visits as the outcome. Both regressions included parent perception, child perception, child age (in months), and child sex as predictors.

## Results

Table 1 shows descriptive data for the full sample, which was 51% male, 4% Hispanic ethnicity, and racially diverse (41% white, 51% African-American/Black, 5% Asian-American, 4% multi-racial or other race). Study participants had a mean age of 95.45 months (*SD* = 7.28 months), and the median parent-reported household income level was in the $60,000-$79,000 range. For all analyses, age was computed in months old at baseline.

Descriptive analyses revealed that parents perceived their child to be able to cross a real street safely without a traffic light slightly more than 50% of the time, on average (*M* = 55.68, *SD* = 28.84). The mean endorsement for a child believing they could cross a street safely without a traffic light was 2.59 (*SD* = 1.50) on average, just above the midpoint of the five-point scale. Examination of the categorical frequency of the responses found that 36.0% of children selected *No*, *definitely not (1)*; 15.4% selected *No*, *probably not (2);* 20.4% selected *Maybe (3);* 10.1% selected *I think so (4);* and 18.1% selected *Yes*, *for sure (5).* This pattern of results suggests that a slight majority of the children endorsed not believing they could cross the street safely (51.4% selected response items one or two, compared to 48.6% who selected response items three to five).

A comparison between boys and girls yielded no statistically significant sex differences for parent or child perceptions of crossing safely. Also non-significant, girls (*M* = 15.70%, *SD* = 9.34%) experienced a slightly higher percentage of unsafe crossings than boys (*M* = 14.25%, *SD* = 9.89%), and girls (*M* = 10.55 training visits, *SD* = 6.44) achieved adult competency slightly more quickly than boys (*M* = 10.75, *SD* = 6.33).

Parent perception of real-world crossings was significantly correlated to children’s baseline unsafe crossings (*r* = −.11, *p*<.05) and number of training visits (*r* = −.14, *p*<.01), but not child perception of real-world crossings, child sex, or child age (see Table 2). Child perception of real-world crossings was not significantly associated with child age, sex, unsafe crossings, or number of trainings. Child age was significantly associated with unsafe crossings and number of training visits, such that older children had fewer unsafe crossings (*r* = −.18, *p*<.01) and fewer training visits (*r* = −.15, *p*<.01).

Results from a multivariable linear regression model (see Table 3) demonstrated significant associations (*F* = 6.44, R^2^ = 0.05) between baseline unsafe crossings and both parent perception of real-world crossings (B = −0.00045, β=−0.10771, *p*<.05, 95%CI [−0.00082,−0.00008]) and child age (B = −0.00310, β=−0.18288, *p*<.01, 95%CI [−0.00446,−0.00153]). Child perception and sex were not significant contributors in the model.

A second linear regression model (see Table 3) examining the number of training visits required to achieve adult competency with the same independent variables was also significant (*F* = 4.52, R^2^ = 0.04),with parent perception of real-world crossings (B = −0.03025, β=−0.13499, *p*<.01, 95%CI [−0.05114,−0.00937]) and child age (B = −0.12144, β=−0.13927, *p*<.01, 95%CI [−0.20267,−0.04022]) again emerging as significant predictors.

## Discussion

We examined parents’ and children’s perceptions of children’s pedestrian abilities and found that parents’ perceptions aligned with their child’s actual pedestrian ability, as measured by both baseline unsafe crossings and number of training visits needed to achieve adult competency. Children’s perceptions of their own pedestrian ability did not align as closely with our study outcomes. Replicating previous research (e.g., Schwebel et al. 2024a, b), child age was associated with their actual pedestrian ability, with small effect sizes. Child sex was not associated with ability or perception.

As hypothesized, parent perceptions were significantly associated with their child’s pedestrian skills. Effect sizes for these associations were small. This is a key finding because parents and other adults serve as one important line of defense to protect children from pedestrian injury, teach children pedestrian skills, and help children learn to cross streets safely on their own. From an intervention standpoint, these findings point to adults as capable and critical for determining how and when to teach their 7- and 8-year-old children safe street-crossing skills, and for supervising them until they develop the requisite skills to cross streets independently.

Child perceptions were not significantly associated with their actual pedestrian abilities. When examining descriptive statistics, a majority of children reported low levels of perceived pedestrian abilities. The mean for unsafe crossings at baseline was 25%, but even just one unsafe crossing can result in a serious injury. These findings may indicate the complexity of pedestrian safety as a task and reflect the struggle children in this age group might face when judging their pedestrian abilities given their rapidly developing cognitive and perceptual skills required both for safe negotiation of traffic and to estimate their own physical and cognitive skills (Plumert and Schwebel 1997; Schwebel et al. 2024a, b),.

Our results support the broader literature suggesting successful child pedestrian injury prevention must be multifaceted (Bou-Karroum et al., 2022; Dowswell et al., 1996). One aspect of prevention should involve traffic environment modifications that separate children from dangerous traffic. Another aspect, supported by our findings, is adult supervision of street-crossing among young children, who have underdeveloped cognitive, perceptual and motor skills, and who often lack extensive experience with and training in pedestrian safety. Finally, consistent with UNCRC Article 12 (United Nations, 1989), children can be trained to cross streets safely and independently. There is evidence that children can develop ability to become safe pedestrians through extensive training by age 7 and 8 (Schwebel et al. 2024a, b). To achieve this, parents and children should collaborate to actively discuss pedestrian safety beginning at a young age, with children given the opportunity to share questions, worries, and expectations related to crossing the street independently and parents listening to and facilitating developmentally appropriate teaching of the skills. Extensive practice, either in virtual or real-world settings, is essential.

Ideal interventions might be multifaceted and tailored to different groups of children, providing tailored training through different strategies depending on children’s needs and individual differences. For children who tend to overestimate their own ability, there is risk that they may choose to cross streets independently before they possess the requisite cognitive, perceptual, and motor abilities to do so. Pedestrian safety interventions for these children might prioritize didactics related to skill development as well as behavioral control and efforts to alter perceived vulnerability to injury. A different set of children may be technically competent at crossing the street safely on their own but underestimate their abilities, perhaps due to lack of self-efficacy or fear. This behavior pattern can lead to delays entering safe traffic gaps, placing children at risk of injury despite their competence to cross safely (Juzdani et al., 2020; Shen et al., 2015; Wang et al., 2020). Interventions for these children might consider exposure-based interventions to reduce fear, emotion regulation strategies to help with self-regulation and self-efficacy, and confidence building approaches.

Broadly, our findings add to the nuanced literature offering mixed findings on the role of parent and child perception in children’s fundamental movement skills (De Pasquale et al., 2021; Maher et al., 2018; Morrongiello et al., 2014; Stanley & Moran, 2017). Some previous research shows patterns that differ from our results, suggesting children’s perceptions are more aligned with their own actual abilities compared to their parents’. These results tend to emerge with complex motor movement skills, such as swimming ability (De Pasquale et al., 2021). Pedestrian behavior requires some motor skills but is most dominated developmentally by cognitive and perceptual abilities (e.g., attention to traffic, judging traffic speeds and distances, identifying safe routes). Children may have appropriate and accurate understanding of their physical motor abilities at age 7 and 8 – “I can get across the street quickly.” – but struggle with metacognitive judgment of their ability for relevant cognitive elements, such as “I can judge a safe traffic gap to cross within.”

This interpretation is consistent with findings from Meir and colleagues (2013), who investigated child and adult pedestrians’ perception of hazards in a crossing decision task. Compared to 9- to 10-year-olds and 10- to 13-year-olds, 7- to 9-year-olds in the study tended to cross more frequently, faster, and in a manner that was consistent with experienced adult pedestrians. However, the experienced adult pedestrians in the study demonstrated greater awareness of potential hazards, indicating informed decision-making among adults when crossing. In contrast, the 7- to 9-year-olds displayed spontaneous responses of fast crossing, primarily citing approaching vehicles as a criterion for crossing rather than acknowledging other hazards in the environment. In the context of our study results, we might infer that parents, as experienced adult pedestrians, recognize that even if their child can physically move quickly, they will struggle with the cognitive-perceptual task of recognizing hazards in the traffic environment. Thus, parents might possess a more comprehensive understanding of their 7- and 8-year-old children’s pedestrian safety abilities than the children, with still-developing cognitive skill, do themselves.

Consistent with previous reports (e.g., Bart et al., 2008; Morgan et al., 2023; Wang et al., 2018), child age was modestly associated with pedestrian behaviors, with older children performing more safely. Age was not associated with parent or child perception of abilities, however, replicating previous literature (Babic et al., 2014; den Uil et al., 2023; Washburn & Kolen, 2018), although the finding from our study should be interpreted cautiously given the fairly narrow age range studied and the fact that children were younger than many experts recommend to engage in independent street-crossing (Kendi et al., 2023). Age might emerge as a more impactful factor if sampling spanned a broader age range, as 7 to 11 years of age is identified in prior research and theory as the most formative stage for the development of safe road crossing skills (Meir et al., 2013).

Study results have several implications for pedestrian injury prevention. First, the finding that children’s perceptions of their abilities did not always align with their actual abilities introduces potential safety concerns. Children in this age group may rely on their own perceived ability to determine which streets to cross independently, and when. Our findings suggest some children may decide to cross before they are developmentally ready to do so safely, or that they restrain from crossing even if they are prepared to do so independently. Youth-informed efforts, either in collaboration with adults in their family or through community- or school-level programs, can teach pedestrian safety skills, increase children’s awareness of their actual pedestrian abilities, and strengthen self-efficacy and self-regulation in the context of street-crossing. They might also incorporate environmental and/or adult behavioral strategies to reduce child pedestrian injury risk.

Second, educating parents on the importance of carefully evaluating children’s skills and teaching children pedestrian safety, even if the child expresses confidence in their own abilities, may mitigate risk. Third, our findings suggest that incorporating assessments of child and especially parent perception in pediatric pedestrian safety training programs could be worthwhile. Fourth, aligned with the focus of UNCRC (United Nations, 1989), incorporating youth voices in intervention development is essential and likely to be impactful.

## Strengths and Limitations

This study offered several methodological strengths, including a large sample size, use of virtual reality to assess children’s pedestrian behavior ethically, and data collection from both children and parents. The study also had limitations. First, our sample was restricted to seven- and eight-year-old children, narrowing variance to detect age-related differences and restricting generalizability to other age groups. Further, our sample excluded children with physical or mental impairments that would invalidate participation in the study, so findings may not generalize to all seven- and eight-year-old children. Second, both child and parent perception data were collected through single self-report items, which introduces potential response bias in interpreting the questions asked and attempting to present children in a positive manner. Moreover, there was a discrepancy in the measurement scales, impacting comparative claims between the items. The parent item was continuous and based on a percentage-based probability estimate whereas the child item was answered on an ordinal five-point scale. Third, the perception questions referred to “real-world” crossing behavior and required children to imagine being in a situation they might not have been familiar with, but the outcome captured crossing behavior in a virtual environment. Outcomes in a simulated environment simplify real-world traffic complexity, social context, and emotional influences. Previous research demonstrates the validity of pedestrian behavior in a virtual world compared to the real world (Schwebel et al., 2008), but future research might replicate findings with real-world pedestrian behavior assessed in an ethical manner. Future studies should replicate these findings using real-world or hybrid observational methods to strengthen ecological validity.

## Conclusions

Pediatric pedestrian injury is a critical public health concern. Our findings address an understudied topic – parent and child perceptions about children’s pedestrian safety skills. The results suggest that parent perception of children’s ability significantly relates to child pedestrian ability, though with small effect sizes. In contrast, child perception relates less consistently and non-significantly to child pedestrian ability. Given the complexity of child perception and pedestrian abilities and the rapid and natural development in cognitive, perceptual, and motor skills relevant to pedestrian safety during middle childhood, prioritizing youth voices may introduce innovative and effective strategies to help children be active agents in becoming safe pedestrians. Further, educating parents concerning child pedestrian safety risks is vital to helping to keep children safe.

## Figures and Tables

**Table 1:** Demographic characteristics of the sample, perception variable outcomes, and primary outcomes, N (%), or mean (SD)

Variable	Sample (*n* = 497)
Child age (months)	95.45 (7.28)
*Child sex*	
Male	233 (46.6%)
Female	264 (52.8%)
*Child ethnicity (n missing = 4)*	
Hispanic	18 (3.6%)
Non-Hispanic	475 (95.0%)
*Child race (n missing = 7)*	
African American/Black	254 (50.8%)
Asian American	24 (4.8%)
Multiracial	12 (2.4%)
Native American	1 (0.2%)
White	199 (39.8%)
*Household income (n missing = 64)*	
<$20,000	69 (15.9%)
$20,000-$39,000	66 (15.2%)
$40,000-$59,000	57 (13.2%)
$60,000-$79,000	51 (11.8%)
$80,000-$99,000	67 (15.5%)
$100,000-$119,000	38 (8.8%)
$120,000-$139,000	45 (10.4%)
$140,000+	40 (9.2%)
Parent perception (%; n missing = 25)	55.68 (28.84)
Child perception (n missing = 3)	2.59 (1.50)
1 – No, definitely not	178 (36.0%)
2 – No, probably not	76 (15.4%)
3 – Maybe	101 (20.4%)
4 – I think so	50 (10.1%)
5 – Yes, for sure	89 (18.1%)
Percentage of unsafe crossings (n missing = 39)	25.72 (12.02)
Number of training visits (n missing = 3)	10.64 (6.38)

**Table 2:** Pearson and point-biserial correlation coefficients between demographic and primary variables

Variable	1	2	3	4	5	6
1. Unsafe crossings	-					
2. Number of trainings	.22**	-				
3. Child age^a^	−.18**	−.15**	-			
4. Child sex^b^	−.11	.02	.01	-		
5. Parent perception	−.11*	−.14**	.02	.02	-	
6. Child perception	−.03	.04	.01	−.06	−.03	-

* Correlation is significant at the *p* <.05 level

** Correlation is significant at the *p* <.01 level

a Age is measured in months old at baseline

b Spearman coefficient, females coded as 0, males coded as 1

**Table 3:** Regression analyses predicting unsafe crossings and number of training visits

Variable	B	β	95% CI		*p*
	
			LL	UL	
*Unsafe crossings*					
Parent perception^a^	−.00045	−.10771	−0.00082	−0.00008	.01764
Child perception	.00066	.00829	−0.00647	0.00780	.85497
Child age^b^	−.00310	−.18288	−0.00446	−0.00153	.00006
Child sex^c^	−.01846	−.07673	−0.03986	0.00295	.09087
*Number of training visits*					
Parent perception	−.03025	−.13499	−0.05114	−0.00937	.00462
Child perception	.16425	.03845	−0.23486	0.56337	.41902
Child age^a^	−.12144	−.13927	−0.20267	−0.04022	.00347
Child sex^b^	.35398	.02763	−0.84254	1.55049	.56122

Decimal values are extended five places to assist with interpretability

a Age is measured in months old at baseline

b Females coded as 0, males coded as 1

## Data Availability

The data that support the findings of this study are available from the corresponding author upon reasonable request. The corresponding author takes full responsibility for the integrity of the data and the accuracy of the data analysis.

## References

[R1] Babic MJ, Morgan PJ, Plotnikoff RC, Lonsdale C, White RL, & Lubans DR. (2014). Physical activity and physical self-concept in youth: Systematic review and meta-analysis. Sports Medicine, 44, 1589–1601. 10.1007/s40279-014-0229-z 25053012

[R2] Bart O, Katz N, Weiss PL, & Josman N. (2008). Street crossing by typically developed children in real and virtual environments. OTJR: Occupation Participation and Health, 28(2), 89–96. 10.3928/15394492-20080301-01

[R3] Barton BK, & Schwebel DC. (2007). The influences of demographics and individual differences on children’s selection of risky pedestrian routes. Journal of Pediatric Psychology, 32(3), 343–353. 10.1093/jpepsy/jsl009 16801325

[R4] Bou-Karroum L, El-Jardali F, Jabbour M, Harb A, Fadlallah R, Hemadi N, & Al-Hajj S. (2022). Preventing unintentional injuries in school-aged children: A systematic review. Pediatrics, 149(Supplement 6), Article e2021053852J. 10.1542/peds.2021-053852J PMC12476578 35503333

[R5] Centers for Disease Control and Prevention (2025). WISQARS injury data. https://wisqars.cdc.gov

[R6] Children and Young People’s Commissioner Scotland. (n.d.). Article 12. https://www.cypcs.org.uk/rights/uncrc/articles/article-12/

[R7] De Pasquale C, De Sousa Morgado L, Jidovtseff B, De Martelaer K, & Barnett LM. (2021). Utility of a scale to assess Australian children’s perceptions of their swimming competence and factors associated with child and parent perception. Health Promotion Journal of Australia, 32(Suppl. 2), 106–115. 10.1002/hpja.404 33462929

[R8] den Uil AR, Janssen M, Busch V, Kat IT, & Scholte RHJ. (2023). The relationships between children’s motor competence, physical activity, perceived motor competence, physical fitness and weight status in relation to age. PLoS ONE, 18(4), Article e0278438. 10.1371/journal.pone.0278438 PMC10104338 37058506

[R9] Dowswell T, Towner EM, Simpson G, & Jarvis SN. (1996). Preventing childhood unintentional injuries–what works? A literature review. Injury Prevention, 2(2), 140–149. 10.1136/ip.2.2.140 9346079 PMC1067679

[R10] Field SC, Foley JT, Naylor PJ, & Temple VA. (2024). Perceptions matter! Active physical recreation participation of children with high and low actual and perceived physical competence. International Journal of Environmental Research and Public Health, 21(9), Article 1129. 10.3390/ijerph21091129 PMC11431184 39338012

[R11] Huynh HT, Demeter NE, Burke RV, & Upperman JS. (2017). The role of adult perceptions and supervision behavior in preventing child injury. Journal of Community Health, 42(4), 649–655. 10.1007/s10900-016-0300-9 28042643

[R12] Juzdani MH, Morgan CH, Schwebel DC, & Tabibi Z. (2020). Children’s road-crossing behavior: Emotional decision making and emotion-based temperamental fear and anger. Journal of Pediatric Psychology, 45, 1188–1198. 10.1093/jpepsy/jsaa076 32951057 PMC7850000

[R13] Kendi S, Johnston BD, Hoffman B, Agran PF, Culyba A, Dodington J, & Kozial B. (2023). Child pedestrian safety. Pediatrics, 152(1), e2023062506. 10.1542/peds.2023-062506 37337837

[R14] Lineweaver TT, Collins AN, Stopa MM, Horth MS, Fishbaugh ME, Haut J, & Busch RM. (2022). Mother knows best… or does she? Perceptions of the memory abilities of pediatric patients with epilepsy as reported by patients and their parents across time. Epilepsy & Behavior, 128, 108589. 10.1016/j.yebeh.2022.108589 35182849

[R15] Liong GH, Ridgers ND, & Barnett LM. (2015). Associations between skill perceptions and young children’s actual fundamental movement skills. Perceptual and Motor Skills, 120(2), 591–603. 10.2466/10.25.PMS.120v18x2 25706343

[R16] Little RJA. (1988). A test of missing completely at random for multivariate data with missing data. Journal of the American Statistical Association, 83(404), 1198–1202.

[R17] Maher SJ, Schott N, Lander NJ, Hinkley T, & Barnett LM. (2018). A comparison of parent report and actual motor competence in young children. Australian Occupational Therapy Journal, 65(5), 387–394. 10.1111/1440-1630.12486 29926927

[R18] Meir A, Parmet Y, & Oron-Gilad T. (2013). Towards understanding child pedestrians’ hazard perception abilities in a mixed reality dynamic environment. Transportation Research Part F: Traffic Psychology and Behaviour, 20, 90–107. 10.1016/j.trf.2013.05.004

[R19] Morano M, Bortoli L, Ruiz MC, Campanozzi A, & Robazza C. (2020). Actual and perceived motor competence: Are children accurate in their perceptions? PLoS ONE, 15(5), Article e0233190. 10.1371/journal.pone.0233190 PMC7219767 32401796

[R20] Morgan CH, Stager LM, Schwebel DC, & Shen J. (2023). A systematic review and meta-analysis on the efficacy of virtual reality pedestrian interventions to teach children how to cross streets safely. Journal of Pediatric Psychology, 48(12), 1003–1020. 10.1093/jpepsy/jsad058 37665734 PMC10733733

[R21] Morrongiello BA, & Barton BK. (2009). Child pedestrian safety: Parental supervision, modeling behaviors, and beliefs about child pedestrian competence. Accident Analysis & Prevention, 41(5), 1040–1046. 10.1016/j.aap.2009.06.017 19664443

[R22] Morrongiello BA, & Corbett M. (2015). Using a virtual environment to study child pedestrian behaviours: A comparison of parents’ expectations and children’s street crossing behaviour. Injury Prevention, 21(5), 291–295. 10.1136/injuryprev-2014-041508 25825352

[R23] Morrongiello BA, Sandomierski M, & Spence JR. (2014). Changes over swim lessons in parents’ perceptions of children’s supervision needs in drowning risk situations. Health Psychology, 33(7), 608–615. 10.1037/a0033881 23977872

[R24] Niemistö D, Barnett LM, Cantell M, Finni T, Korhonen E, & Saakslahti A. (2022). What factors relate to three profiles of perception of motor competence in young children? Journal of Sports Sciences, 40(2), 215–225. 10.1080/02640414.2021.1985774 34636285

[R25] O’Neal EE, Jiang Y, Franzen LJ, Rahimian P, Yon JP, Kearney JK, & Plumert JM. (2018). Changes in perception–action tuning over long time scales. Journal of Experimental Psychology: Human Perception and Performance, 44(1), 18–26. 10.1037/xhp0000378 28425731 PMC8715675

[R26] Pesce C, Masci I, Marchetti R, Vannozzi G, & Schmidt M. (2018). When children’s perceived and actual motor competence mismatch. Journal of Motor Learning and Development, 6(Suppl. 2), S440–S460. 10.1123/jmld.2016-0081

[R27] Plumert JM. (1995). Relations between children’s overestimation of their physical abilities and accident proneness. Developmental Psychology, 31(5), 866. 10.1037/0012-1649.31.5.866

[R28] Plumert JM, & Schwebel DC. (1997). Social and temperamental influences on children’s overestimation of their physical abilities: Links to accidental injuries. Journal of Experimental Child Psychology, 67(3), 317–337. 10.1006/jecp.1997.2411 9440296

[R29] Putnick DL, Hahn CS, Hendricks C, Suwalsky JTD, & Bornstein MH. (2020). Child, mother, father, and teacher beliefs about child academic competence. Journal of Research on Adolescence, 30, 298–314. 10.1111/jora.12477 PMC6697633 30771240

[R30] Riaz MS, Cuenen A, Polders E, Akram MB, Houda M, Janssens D, & Azab M. (2022). Child pedestrian safety: Study of street-crossing behaviour of primary school children with adult supervision. Sustainability, 14(3), Article 1503. 10.3390/su14031503

[R31] Sando OJ, Kleppe R, & Sandseter EBH. (2024). Risk willingness in play: Exploring children’s behaviour in a virtual reality playground. European Early Childhood Education Research Journal. 10.1080/1350293X.2024.2437769

[R32] Schwebel DC, Gaines J, & Severson J. (2008). Validation of virtual reality as a tool to understand and prevent child pedestrian injury. Accident Analysis & Prevention, 40(4), 1394–1400. 10.1016/j.aap.2008.03.005 18606271

[R33] Schwebel DC, Barton BK, Shen J, Wells HL, Bogar A, Heath G, & McCullough D. (2014). Systematic review and meta-analysis of behavioral interventions to improve child pedestrian safety. Journal of Pediatric Psychology, 39(8), 826–845. 10.1093/jpepsy/jsu024 24864275 PMC4138804

[R34] Schwebel DC, Johnston A, McDaniel D, Severson J, He Y, & McClure LA. (2024a). Teaching children pedestrian safety in virtual reality via smartphone: A non-inferiority randomized clinical trial. Journal of Pediatric Psychology, 49, 405–412. 10.1093/jpepsy/jsae020 38637283 PMC11175590

[R35] Schwebel DC, Sando OJ, Sandseter EBH, & Kleppe R. (2024). Age, sex, sensation-seeking, and road-crossing: How does risk context impact children’s street-crossing? Traffic Injury Prevention, 25(7), 986–992. 10.1080/15389588.2024.2358103 38833267

[R36] Shen J, McClure LA, & Schwebel DC. (2015). Relations between temperamental fear and risky pedestrian behavior. Accident Analysis & Prevention, 80, 178–184. 10.1016/j.aap.2015.04.011 PMC10402914 25912314

[R37] Stanley T, & Moran Dr K. (2017). Parental perceptions of water competence and drowning risk for themselves and their children in an open water environment. International Journal of Aquatic Research and Education, 10(1), 4. 10.25035/ijare.10.01.04

[R38] United Nations Office of the High Commissioner for Human Rights (1989, November 20). Convention on the Rights of the Child. https://www.ohchr.org/en/instruments-mechanisms/instruments/convention-rights-child

[R39] Wang H, Schwebel DC, Tan D, Shi L, & Miao L. (2018). Gender differences in children’s pedestrian behaviors. Journal of Safety Research, 67, 127–133. 10.1016/j.jsr.2018.09.003 30553414 PMC6296240

[R40] Wang H, Morgan C, Li D, Huang R, & Schwebel DC. (2020). Children’s fear in traffic and its association with pedestrian decisions. Journal of Safety Research, 76, 56–63. 10.1016/j.jsr.2020.11.010 33653569 PMC8895428

[R41] Washburn R, & Kolen A. (2018). Children’s self-perceived and actual motor competence in relation to their peers. Children, 5(6), 72. 10.3390/children5060072 29890698 PMC6025321

